# Why Not Every Trained Nurse Awake and Active?

**Published:** 1919-01-18

**Authors:** 


### THE AWAKENED COLLEGE.

### Why not Every Trained Nurse Awake and Active?

I am following the suggestion of a correspondent in last week's Hospital, and adopting the title "The Awakened College"—but I am non-committal as to whether the description thus conveyed applies to the present or the future!

### A Spiritual Edifice.

The College of Nursing, as I see it, is a spiritual rather than a material edifice, and the stones thereof consist of human souls. Some day, doubtless, there will be a building of brick and stone—scores of buildings probably—and above the portals will be graven the words "College of Nursing." These buildings will possess a certain utility, but otherwise they are mere inanimate emblems of pulsating life, and when the passing wayfarer reads the sign his thoughts will travel involuntarily to the hospital ward where lie sick and suffering men and women, tended and comforted by nurses whose gentleness, patience, and skill—nay, whose very uniform seems to invest them with the verisimilitude of ministering angels. The College of Nursing is that intangible bond which unites nurses in one great army, and it is probably easier to picture it as a building than as aught else. Is this spiritual College awake? Is it growing upwards, expanding outwards, civilising, ennobling, irradiating, illuminating?

### Let Every Member Examine her Soul.

It is for every member of the College by examining her own soul to answer this question, rather than for me. I do not even know whether the members realise that they are living stones in a great building, or whether they regard the College as a thing of brick and stone, or yet whether it appears to them that the Executive who gather together in London and elsewhere to organise and regulate compose the College. I have no means of appreciating with what clearness of vision they view the landscape. But I am convinced that until they realise the spiritual meaning of the College, and that they are themselves component and indispensable parts of the structure, they cannot accurately be described as awake.

### Where the College is Urgently Needed.

That the establishment of the College was urgently needed in the kingdom of the nurse there is no more striking proof than the strength of its membership. The master builder who is its President has undoubtedly conferred on the profession a benefit which cannot be fully apparent to the nurse this year or next any more than the magnificent organisation of the Red Cross can be apparent to the wounded soldier. He sees a little corner only, and he thanks God for that. But he would require to travel to every battle front, and to every corner of the Empire as well, in order to realise all that is comprehended in the mystic words "the Red Cross." To say that it cost fourteen millions, or that so many men participated in its benefits, or to visualise the miles of hospital trains and ambulance cars is to see as through a glass darkly what has been achieved in four years of war. The Home Front, otherwise the whole civil population, had to be inspired, recruited, organised, and Red Cross money and material were in direct and mathematical proportion to the completeness of this colossal task.

### What the College will Achieve for Nurses.

I pay this tribute to the champion and chairman of the Red Cross because I feel that what he has achieved for our men-at-arms is an earnest and an assurance of what the College will achieve for nurses, always provided that he is supported by those concerned. Personally, I have no hesitation in confessing that I am somewhat of a materialist. I want to see tangible results. The Doctor, the Surgeon, the Nurse, the ambulance train, the bandages, the splints—these are some of the Red Cross tangibles. Behind these things are the moral and the spiritual forces, the motive power. Similarly in the nurse's kingdom I look for substantial results—good pay, short hours, freedom from weariness, freedom from worry should to-morrow bring disablement. I want to see all this developing, just as I had the privilege of witnessing the making of bandages and dressings for wounded fighting men. I am impatient for progress, and I note from the published correspondence on the subject which has reached The Hospital that I am not alone in this respect. There is abundant evidence that many members of the College are both alive and awake.

### The Influence of the Humblest Member.

The one outstanding feature of the College constitution which convinces me of its latent and undeveloped possibilities is that the humblest member, if she chooses, can influence its power and growth. The Executive Committee, its *personnel*, its activity, its energy, its policy—all these depend on the nurse. She is the motor, and if she throbs the Committee will respond. There are "duds" in Parliament, but why are they there? Who is to blame? The answer is, an apathetic electorate. Every College member should be a recruiting agent for additional membership and an active agitator for progress. If there is no "Centre" within reach she should press for one. And above all she should interest herself in the doings of the Executive. Who are the active members, and who the members who are too busily occupied otherwise to take interest in the College? The Executive elections are approaching, and a great deal will depend on the nominees for the ensuing year. If the College is really awake all this should be discussed at local centres, and those who are elected should be persons who in actual fact, and not in name only, represent and voice the nurses.

### Its Power, Its Influence, and Its Strength.

The College, its power, its influence, its strength depends wholly on the number, the vitality, and the will power of its members. My earnest advice to them is to gather in recruits by the thousand, and clamour to have their grievances redressed. The British public will respond to the call without hesitation. Meanwhile there is much reason for satisfaction. The mere fact that there is such a magnificent institution thoroughly established, that it is daily growing numerically and financially is a sure indication of future achievement. The obstacles of opposition are being swept away, and the outlook is bright. Ruskin in his "Seven Lamps of Architecture" points out that the great principle of success in every human effort is comprehended in the words "know what you have to do, and do it." The many failures to which the efforts of intelligent men are liable, he says, spring from the single error of not recognising this. If every member of the College realises the application of this to herself then it cannot be denied that the College is awake.

IERNE.

